# *In vitro* and *in vivo* screening for novel essential cell-envelope proteins in *Pseudomonas aeruginosa*

**DOI:** 10.1038/srep17593

**Published:** 2015-12-01

**Authors:** Regina Fernández-Piñar, Alessandra Lo Sciuto, Alice Rossi, Serena Ranucci, Alessandra Bragonzi, Francesco Imperi

**Affiliations:** 1 Laboratory of Molecular Microbiology, Department of Biology and Biotechnology “Charles Darwin”, Sapienza University of Rome, Rome, Italy; 2 Division of Immunology, Transplantation and Infectious Diseases, San Raffaele Scientific Institute, Milan, Italy; 3 Pasteur Institute-Cenci Bolognetti Foundation, Sapienza University of Rome, Rome, Italy

## Abstract

The Gram-negative bacterium *Pseudomonas aeruginosa* represents a prototype of multi-drug resistant opportunistic pathogens for which novel therapeutic options are urgently required. In order to identify new candidates as potential drug targets, we combined large-scale transposon mutagenesis data analysis and bioinformatics predictions to retrieve a set of putative essential genes which are conserved in *P. aeruginosa* and predicted to encode cell envelope or secreted proteins. By generating unmarked deletion or conditional mutants, we confirmed the *in vitro* essentiality of two periplasmic proteins, LptH and LolA, responsible for lipopolysaccharide and lipoproteins transport to the outer membrane respectively, and confirmed that they are important for cell envelope stability. LptH was also found to be essential for *P. aeruginosa* ability to cause infection in different animal models. Conversely, LolA-depleted cells appeared only partially impaired in pathogenicity, indicating that this protein likely plays a less relevant role during bacterial infection. Finally, we ruled out any involvement of the other six proteins under investigation in *P. aeruginosa* growth, cell envelope stability and virulence. Besides proposing LptH as a very promising drug target in *P. aeruginosa*, this study confirms the importance of *in vitro* and *in vivo* validation of potential essential genes identified through random transposon mutagenesis.

The Gram-negative bacterium *Pseudomonas aeruginosa* is currently regarded as one of the most dreaded opportunistic pathogens in hospitals, and belongs to the “ESKAPE pathogens” group due to its propensity to “escape” antibiotic treatments^1^. *P. aeruginosa* is also the main cause of chronic lung infection and mortality in individuals with cystic fibrosis (CF), which is the most common fatal genetic disease in the Caucasian population^2^. A hallmark of *P. aeruginosa* infections is the life-threatening severity and poor responsiveness to currently available antibiotic therapies^3^. Besides its intrinsic resistance to many widely used antibiotics, that is mostly due to low membrane permeability and active drug efflux, *P. aeruginosa* also managed to acquire resistance via additional mechanisms, including target modification, increased expression of efflux pumps and acquisition of new drug-resistance genes by horizontal gene transfer^4^,^5^. As a result, pan-resistance to currently used antibiotics in clinical *P. aeruginosa* isolates has already been reported^5^,^6^. Despite the growing concern of clinicians about the very limited number of therapeutic options to fight multi-resistant *P. aeruginosa* strains, only a very small number of new anti-*Pseudomonas* drugs are currently in late stage of pre-clinical or clinical development^7^,^8^. In this scenario, there is an impelling need for the identification of novel drug targets and the discovery of new-generation antibiotics active against *P. aeruginosa*.

A whole genome *in silico* study has predicted that about 38% of the *P. aeruginosa* genome (corresponding to more than 2,100 genes) encodes for proteins that are located in the cell envelope or secreted in the extracellular milieu^9^. Gram-negative bacteria have a typical diderm cell organization, with a cell envelope consisting of a peptidoglycan-containing periplasmic space sandwiched between the cytoplasmic (or inner) membrane and the outer membrane, which is an asymmetric bilayer with an inner leaflet of phospholipids and an outer leaflet of lipopolysaccharide (LPS)^10^. Periplasmic and outer membrane proteins are mainly translocated through the cytosplasmic membrane via the general secretory (Sec) or twin-arginine translocation (TAT) pathways, that recognize target proteins by means of specific N-terminal signal peptides^11^.

Cell envelope proteins are involved in different cellular processes, including cell-wall assembly and stability, nutrient uptake, energy production, adherence, motility, environmental sensing, virulence, and antibiotic resistance. In the last decade, several proteomics studies focused on the characterization of the *P. aeruginosa* membrane and periplasmic compartments (reviewed in^12^), revealing that about one third of the *P. aeruginosa* outer membrane and periplasmic proteins cannot be assigned to any functional class on the basis of sequence homology^13^. Taken together, the relevance of the cell envelope for the physiology of Gram-negative bacteria and the poor functional characterization of the cell envelope sub-proteomes of *P. aeruginosa* suggest that the extracytoplasmic compartments of *P. aeruginosa* cells could represent a promising reservoir of still-unexplored protein functions to be investigated as potential targets for drug development.

Two large-scale transposon mutagenesis projects, focused on two different strains of *P. aeruginosa* (PAO1 and PA14), led to the identification of 335 genes shared by the two strains that were not disrupted in more than 60,000 defined transposon insertion mutants^14^,^15^. A probabilistic calculation of 60,000 random insertions over 6.5 megabases (*i.e.* the average size of the *P. aeruginosa* genome) suggested that a gene 327 bp in length should have a 95% probability of being disrupted by random transposon insertion^15^, indicating that undisrupted genes larger than 327 bp are good candidates as potential essential genes.

Based on this information, we retrieved eight candidate essential genes of *P. aeruginosa* (undisrupted in the two transposon mutagenesis studies described above) that (i) are longer than 327 bp, (ii) have not previously been confirmed as essential genes in *P. aeruginosa*, (iii) are present in all *P. aeruginosa* genomes sequenced so far, and (iv) encode proteins with a predicted cell-envelope localization (Table 1). The role of the selected proteins in *P. aeruginosa* growth *in vitro*, cell envelope stability and pathogenicity in different animal models of infection was investigated in order to evaluate their suitability as potential targets for the development of novel anti-*P. aeruginosa* drugs.

## Results

### Generation of deletion and conditional mutants, and *in vitro* growth assays

With the aim of characterizing novel *P. aeruginosa* proteins involved in cell envelope biogenesis and/or homeostasis, we focused our attention on eight genes that were previously proposed to be essential due to the inability to obtain transposon insertion mutants in more than 60,000 defined transposition events^14^,^15^, and that encode proteins with a predicted cell envelope localization (Table 1). Five of these genes encode hypothetical proteins whose function cannot be predicted on the basis of sequence homology (www.pseudomonas.com), while the remaining genes encode a putative c-type cytochrome or homologues of the *Escherichia coli* LolA and LptA proteins, which are responsible for the transport of lipoproteins or LPS across the periplasm to the outer membrane, respectively^16^,^17^. Due to the presence of another gene named *lptA* in the *P. aeruginosa* genome, which encodes a lysophosphatidic acid acyltransferase, the LptA homologue of *P. aeruginosa* has recently been renamed LptH^18^. A further candidate gene which fulfilled our selection criteria, PA3988 (encoding an homologue of the outer membrane protein LptE of *E. coli*), was not included in the screening since it is predicted to be involved in the same LPS transport pathway of LptH^17^.

**Figure 1:**
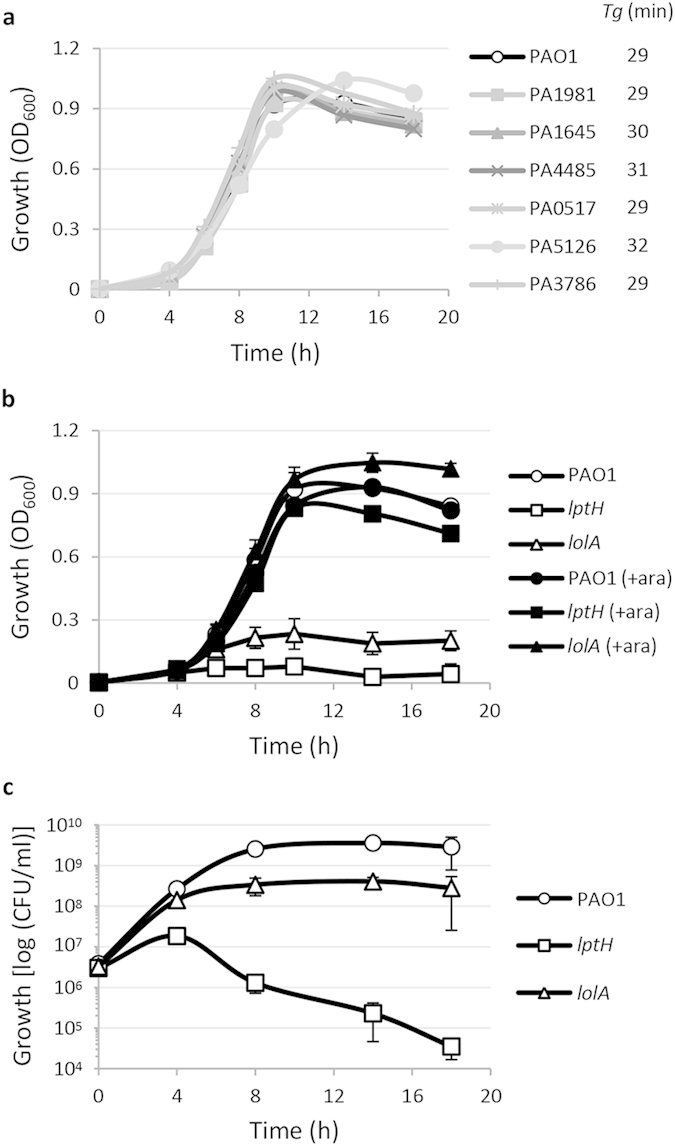
Role of each protein of interest in *P. aeruginosa* growth *in vitro*. (**a**) Growth curves of the wild type strain PAO1 and the PA0517, PA1645, PA1981, PA3786, PA4485 and PA5126 deletion mutants in MH broth at 37 °C in microtiter plates at 200 rpm. The generation time (*Tg*) of each strain is reported in the figure. Results are the mean of three independent experiments, with standard deviations (SD) being <10% of the values. (**b**,**c**) Growth of PAO1 and *lptH* and *lolA* conditional mutants in MH broth at 37 °C in microtiter plates at 200 rpm in the absence or in the presence of 0.5% arabinose (+ara), after a 1:1000 dilution from overnight cultures in MH supplemented with 0.5% arabinose. Growth was measured as OD_600_ (panel b) or CFU/ml (panel c). Results are the mean (±SD) of three independent experiments.

In order to verify the essentiality of the genes of interest, we first attempted to generate unmarked in-frame deletion mutants in the reference strain *P. aeruginosa* PAO1. Surprisingly, we obtained a deletion mutant in six genes of interest (PA0517, PA1645, PA1981, PA3786, PA4485 and PA5126; Table 2), including all the genes encoding proteins with unpredictable function. This evidence clearly rules out that these genes are strictly essential for *P. aeruginosa* growth *in vitro*, as confirmed by the finding that all the deletion mutants showed growth yields and kinetics overall comparable to those of the wild type strain, both in Mueller-Hinton (MH) broth (Fig. 1a) and in MH agar plates (Supplementary Fig. S1), as well as in other complex (LB) or minimal media (M9 supplemented with different carbon sources) (Supplementary Fig. S2).

In contrast, several attempts to generate deletion mutants in *lptH* and *lolA* failed, suggesting that these two genes are important for *P. aeruginosa* growth and/or viability, at least under our experimental conditions. To confirm this hypothesis, we generated *lptH* and *lolA* conditional mutants carrying an arabinose-inducible copy of each gene of interest in a neutral site of the genome and an in-frame deletion in the endogenous gene (Table 2), and assessed their ability to grow in MH broth in the presence or in the absence of arabinose. The growth of both conditional mutants was strongly impaired in MH broth, but was restored to wild type levels in the presence of arabinose (Fig. 1b), confirming the relevance of both LolA and LptH for *P. aeruginosa* growth as well as the reliability of our conditional mutants. Notably, while the *lptH* conditional mutant did not grow at all in the absence of arabinose, the *lolA* conditional mutant showed some residual growth (Fig. 1b). This was confirmed by measuring the number of colony-forming units (CFU) over time. As shown in Fig. 1c, the *lolA* conditional mutant incubated in MH without arabinose was able to perform about six generations before stabilizing at a cell density about 8.5 fold lower than the wild type. In contrast, *lptH* mutant cells only completed a couple of generations in the absence of arabinose before beginning to die (Fig. 1c). Comparable growth profiles were obtained in different complex or minimal media (Supplementary Fig. S2). Overall, these results indicate that, in our experimental conditions, LptH is essential for both *P. aeruginosa* growth and cell viability, while LolA only appears to be important for growth.

### Cell envelope stability

**Figure 2:**
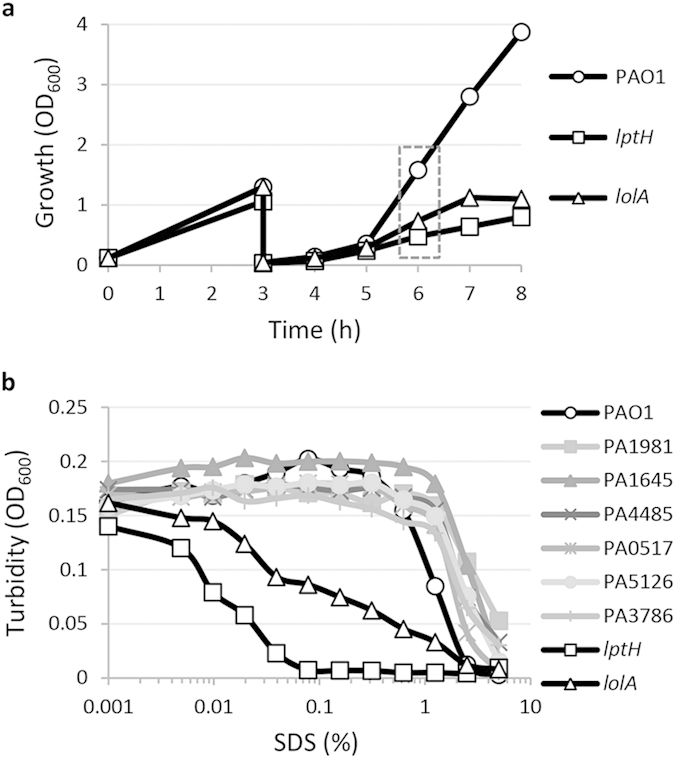
Effect of depletion of each protein of interest on cell envelope stability. (**a**) Growth of PAO1 and the *lptH* and *lolA* conditional mutants at 37 °C in MH broth at 200 rpm in flasks after two successive subcultures in the absence of arabinose, in order to obtain cells depleted of the LptH or LolA protein. Bacteria were cultured for 14 h at 37 °C and 200 rpm in MH supplemented with 0.1% (PAO1 and the *lolA* conditional mutant) or 0.5% arabinose (*lptH* conditional mutant) (not shown in the figure) and then diluted 1:30 in fresh medium without arabinose (time 0). After 3 h of growth, cultures were diluted again 1:30 in fresh medium and incubated at 37 °C until the appearance of a growth defect in the conditional mutants with respect to the wild type. (**b**) Lytic effect of different SDS concentrations (0–5%), measured as decrease in cell suspension turbidity (OD_600_), on PAO1 wild type cells, the PA0517, PA1645, PA1981, PA3786, PA4485 and PA5126 deletion mutant cells, and the LptH- or LolA-deficient conditional mutant cells (*lptH* and *lolA*, respectively) cultured as shown in panel a. The graphs are representative of three independent experiments giving similar results.

Since all the genes of interest encode proteins that are predicted to be located in the cell envelope or, in the case of PA1981, secreted into the extracellular milieu (Table 1), the possible role of these proteins in cell envelope biogenesis and/or stability was assessed through a sodium dodecyl sulphate (SDS) sensitivity assay. Deletion mutants were grown in MH, washed once with saline, and resuspended in saline in the presence of increasing concentrations of SDS. Since the expression of the LptH and LolA proteins was found to be essential for the growth of the *P. aeruginosa lolA* and *lptH* conditional mutants *in vitro* (Fig. 1), a previously-developed culturing strategy was employed to obtain *P. aeruginosa* cells depleted of each protein of interest^19^. Briefly, the *lptH* and *lolA* conditional mutants were grown in MH in flasks for 14 h in the presence of 0.5% (*lptH* mutant) or 0.1% arabinose (*lolA* mutant), and then two successive refreshes (1:30 dilution) were performed in the absence of arabinose. As soon as a growth defect was observed in the conditional mutants with respect to wild type cultures (dashed box in Fig. 2a), cells were collected and tested for SDS sensitivity. All deletion mutants showed an SDS sensitivity profile comparable to that of the wild type strain PAO1 (Fig. 2b). On the other hand, LptH- and LolA-deficient cells were much more sensitive to the lytic effect of SDS, although the defect in SDS resistance was more pronounced in the *lptH* conditional mutant compared to the *lolA* conditional mutant (Fig. 2b). Notably, SDS resistance was restored to wild type levels in both mutants when they were cultured in the presence of 0.5% arabinose (Supplementary Fig. S3). In the whole, this experiment demonstrates that LptH and, to a lesser extent, LolA are the only proteins here investigated with a relevant role in cell envelope stability.

### Pathogenicity in *Galleria mellonella*

**Figure 3:**
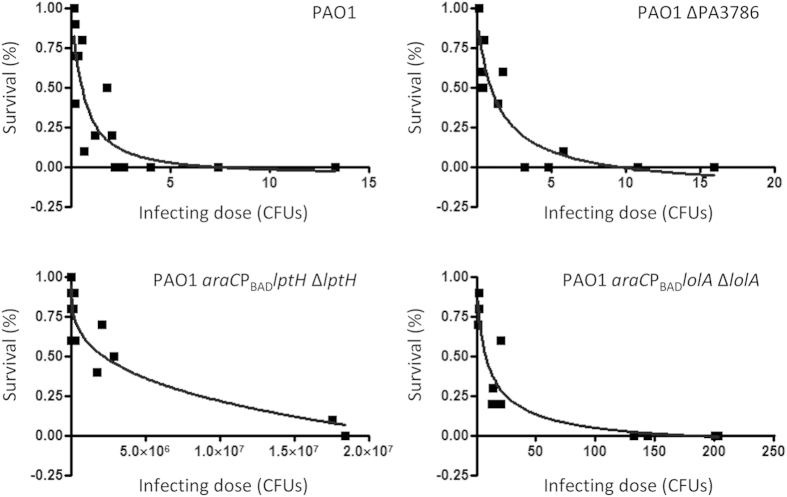
Pathogenicity of selected *P. aeruginosa* mutants in the *G. mellonella* infection model. Survival curves, generated by the GraphPad Prism software, of *G. mellonella* larvae infected with different doses of *P. aeruginosa* PAO1, the *lolA* and *lptH* conditional mutants, and a representative deletion mutant (PAO1 ΔPA3786). The survival curves for the remaining deletion mutants are shown in Supplementary Fig. S4.

Since laboratory cultures could not reflect bacterial growth during infection, each deletion or conditional mutant was also tested in a model of infection based on the larvae of the insect *G. mellonella*, which represents a convenient and easy-to-handle animal model to screen the pathogenicity of *P. aeruginosa* mutants^20^. All the deletion mutants showed lethality curves similar to that of the wild type strain (Fig. 3 and Supplementary Fig. S4), indicating that none of them was relevantly impaired in pathogenicity in this model of infection. This was confirmed by a less-than-2-fold change between the lethal dose 90% (LD_90_) values of mutants and wild type, with the only exception of PAO1 ΔPA1981, whose LD_90_ was 2.5-fold higher than that of PAO1 (Table 3). In agreement with the results of the growth assays, the *lptH* and *lolA* conditional mutants were found to be significantly impaired in infectivity in *G. mellonella* larvae, although a remarkable difference in the pathogenic behaviour of the two strains was observed. Indeed, while the *lptH* conditional mutant showed an LD_90_ of 1.6 × 10^7^ cells, in line with that recently determined in the same model of infection for *P. aeruginosa* conditional mutant cells lacking the essential periplasmic protein TolB^19^, the LD_90_ of the *lolA* conditional mutant was about 60 cells, less than 25-fold higher than that of the wild type (Table 3). To verify that the observed lethality of the *lolA* mutant was related to active growth *in vivo* rather than to increased toxicity of LolA-depleted cells, we infected five *G. mellonella* larvae with about 500 cells of the wild type PAO1 and the *lolA* conditional mutant, and determined the number of viable cells in the hemolymph of dead larvae on selective plates supplemented or not with 0.5% arabinose. The number of viable cells per larva determined on arabinose-containing plates was comparable between the wild type and the *lolA* conditional mutant [1.6 (±0.3) × 10^10^ and 2.1 (±0.4) × 10^10^, respectively)], while the number of CFU on plates without arabinose was below the detection limit of the assay (250 cells) for *lolA*-infected larvae. Overall, these results indicate that, although impaired in virulence with respect to the wild type, the *lolA* conditional mutant is still able to grow and cause infection in *G. mellonella*, suggesting that LolA depletion could be less important for *P. aeruginosa* growth *in vivo* than that observed in selected laboratory media.

### Pathogenicity in a mouse model of pulmonary infection

**Figure 4:**
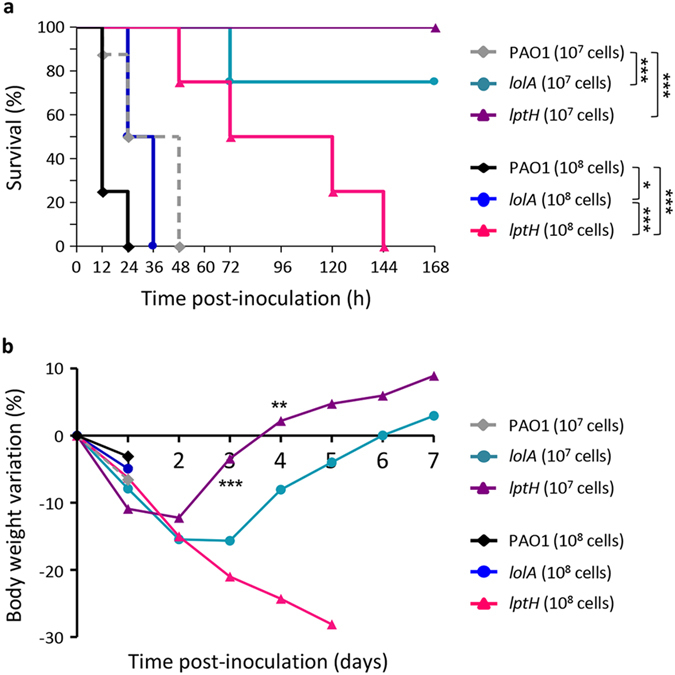
Pathogenicity of *lptH* and *lolA* conditional mutants in a mouse lung infection model. (**a**) Mortality and (**b**) body weight curves for mice (n = 8) infected with 10^7^ or 10^8^ cells of PAO1, the *lptH* or the *lolA* conditional mutant, previously cultured in TSB supplemented with 0.5% arabinose. Data were pooled from two independent experiments. *P < 0.05; **P < 0.01; ***P < 0.001. The asterisks in panel b refer to the differences between mice infected with 10^7^ cells of the *lptH* and *lolA* conditional mutants.

Considering the different behaviour of the *lptH* and *lolA* conditional mutants in the *G. mellonella* infection model, we assessed their pathogenicity in a mouse model of acute lung infection^21^, in order to further verify their suitability as anti-*P. aeruginosa* drug targets. A preliminary experiment revealed that a dose corresponding to 10^7^ cells of our wild type strain was necessary to cause 100% lethality in this infection model (Supplementary Fig. S5). We then compared the pathogenicity of the wild type and each conditional mutant at two different infecting doses (10^7^ and 10^8^ cells). As expected, all mice infected with 10^7^ cells of the wild type strain died within 48 h post infection (Fig. 4a). In contrast, at the same infecting dose, the *lolA* conditional mutant only caused lethality in 25% of mice, while the *lptH* conditional mutant was completely avirulent (Fig. 4a). Moreover, a significant difference in body weight recovery after the infection was observed between mice infected with the two conditional mutants (Fig. 4b), suggesting that the challenge with the *lptH* conditional mutant had a less deleterious effect on mice as compared to that with the *lolA* conditional mutant. This was also confirmed by the observation that very high infecting doses (10^8^ cells) caused 100% mortality within 36 h from the challenge in the case of the *lolA* conditional mutant, while mice infected with the *lptH* conditional mutant showed a significantly delayed mortality curve (Fig. 4a).

## Discussion

The rise of antibacterial resistance among bacterial pathogens leads to a growing need for the identification and development of novel antibacterial agents. Notably, the recent discovery of new promising antimicrobials with a completely new mechanisms of action^22^,^23^ indicates that we have only explored a fraction of the microbial targets that could be used for antibiotic drug discovery.

Rational development of antibacterial drugs with novel mechanisms of action requires the identification of new molecular targets, that may emerge from a better understanding of cellular processes essential for cell survival and/or pathogenicity. In this view, large-scale systematic analysis of gene essentiality represented an important step towards the characterization of novel potential drug targets. High density whole-genome transposon mutagenesis followed by sequence-based identification of insertion sites was the most frequently used approach to predict gene essentiality in bacterial species (http://www.essentialgene.org/). However, transposon mutagenesis suffers from intrinsic biases that can lead to misannotation of some essential or non-essential genes^24^. Moreover, since gene essentiality prediction by random transposon mutagenesis relies on the inability to obtain mutants in a given gene, this approach does not allow to get information about the function of each putative essential protein or its suitability as a potential antibiotic target.

The present study was specifically aimed at verifying the *in vitro* and *in vivo* essentiality of selected *P. aeruginosa* cell-envelope proteins, in order to propose them as novel potential drug targets. The rationale for focusing on cell envelope-located or surface-exposed proteins is linked to the fact that they are expected to be more accessible to drugs, and drug binding to cell envelope targets could avoid or delay later extrusion by efflux pumps, which represent key components of both intrinsic and acquired antibiotic resistance in *P. aeruginosa*^25^. Among the eight genes here investigated, only two were found to be required for *P. aeruginosa* growth *in vitro*, namely *lptH* and *lolA* (Fig. 1), responsible for the transport of LPS and lipoproteins to the outer membrane, respectively. In agreement with the predicted role of these proteins in outer membrane biogenesis, LptH- and LolA-depleted cells also showed increased sensitivity to the detergent SDS (Fig. 2). In contrast, the remaining genes (PA0517, PA1645, PA1981, PA3786, PA4485 and PA5126) appeared to be dispensable for *P. aeruginosa* growth *in vitro* and for cell envelope stability (Figs 1 and 2). This finding is in line with very recent Tn-seq studies reporting that transposon insertion mutants in these genes were able to grow in different growth media^26^,^27^. Here we also demonstrate that the deletion of each of these genes has no relevant impact on *P. aeruginosa* pathogenicity in the *G. mellonella* model of infection (Fig. 3, Supplementary Fig. S4 and Table 3), clearly ruling out the corresponding proteins as potential targets for conventional antimicrobial drugs.

Notably, the *G. mellonella* infection model also revealed a different pathogenic behaviour of the *lolA* and *lptH* conditional mutants. Indeed, while LptH depletion almost completely abrogated *P. aeruginosa* infectivity in *G. mellonella* larvae, LolA depletion only resulted in a 25-fold increase in the LD_90_ (Fig. 3 and Table 3). A similar trend was also observed in a mouse model of lung infection, although the high infecting doses required to establish the infection in this model likely mitigated the differences between the two conditional mutants, as well as between conditional mutants and the wild type strain (Fig. 4). One could hypothesize that small amounts of arabinose possibly present in *G. mellonella* hemolymph or in the mouse lung could trigger sufficient *lolA* expression to promote growth of the arabinose-dependent conditional mutant. While to the best of our knowledge arabinose levels in *G. mellonella* larvae have not been investigated to date, arabinose concentration was found to be ≤0.02 mg/ml in mouse serum^28^ (corresponding to 0.002%), and should reasonably be even lower in the lung. This arabinose concentration is far below that required to induce growth of the *lolA* conditional mutant *in vitro* (Supplementary Fig. S6), indirectly ruling out that the observed pathogenicity *in vivo* may be due to arabinose-dependent restoration of LolA expression. This is further corroborated by the different pathogenic behaviour in animal models observed for the *lptH* conditional mutant, which carries the same arabinose-dependent regulatory element and shows an *in vitro* response to increasing arabinose concentrations overall comparable to the *lolA* mutant (Supplementary Fig. S6).

Although the inability to obtain a *lolA* deletion mutant does not allow to definitely conclude that LolA, even at low intracellular levels, is not required for *P. aeruginosa* virulence, the residual pathogenicity of the *lolA* conditional mutant in animal models suggests that *P. aeruginosa* cells are viable and can grow *in vivo* with an impaired transport of lipoproteins to the outer membrane. By combining all the whole-genome transposon mutagenesis data available so far^14^,^15^,^26^,^27^,^29^, we found that only one of the 135 putative outer membrane lipoproteins of *P. aeruginosa*^30^ was predicted to be essential for *in vitro* growth in all experimental conditions tested. This protein corresponds to the *E. coli* LolB homologue (PA4668), which is responsible for lipoprotein anchoring into the outer membrane^31^,^32^. However, the above-mentioned transposon mutagenesis studies also predicted *lolA* as an essential gene in *P. aeruginosa*^14^,^15^,^26^,^27^,^29^, in agreement with our *in vitro* results in laboratory media (Fig. 1 and Fig. S2), suggesting that the growth conditions tested in those studies could not be suitable to appreciate the residual growth and infectivity of LolA-depleted cells that we observed *in vivo*. Although further studies are clearly required to characterize at the biochemical and ultrastructural level the outer membrane of LolA-depleted cells during *in vivo* growth, as well as to decipher the *in vivo* factor(s) associated with the restored growth of the *lolA* conditional mutant during infection, from a clinical point of view our findings argue against LolA as an ideal target for anti-*P. aeruginosa* drug development, since LolA inhibition could only partially affect *P. aeruginosa* growth and persistence during infections. Notably, while the essential role of *E. coli* LolA for *in vitro* growth is well known since many years^33^, and its mechanism of action has been investigated in great detail^34^, the infectivity of *E. coli* cells depleted of LolA has never been assessed in animal models. Thus, considering that our *in vivo* results provide evidence of partially-retained infectivity of *P. aeruginosa* cells impaired in lipoproteins transport, it would be interesting to verify the pathogenic role of LolA both in the model bacterium *E. coli* and in other Gram-negative bacteria in which *lolA* homologues have been predicted to be essential by *in vitro* large-scale mutagenesis studies (e.g., *Vibrio*, *Haemophilus, Acinetobacter*, *Burkholderia, Helicobacter*; http://www.essentialgene.org/).

In contrast, LptH depletion in *P. aeruginosa* resulted in growth inhibition and loss of cell viability *in vitro* (Fig. 1), as well as in almost complete abrogation of the ability to cause infection in different animal models (Table 3 and Fig. 4). Notably, an inhibitor of LPS transport in *P. aeruginosa* has already been reported^35^. This peptidomimetic blocks LPS translocation across the outer membrane by inhibiting the activity of the outer membrane protein LptD^36^, and was demonstrated to prevent *P. aeruginosa* lethality in a mouse septicemia model^35^. The *in vitro* and *in vivo* results obtained here with the *lptH* conditional mutant, together with the recently-disclosed three-dimensional structure of *P. aeruginosa* LptH^18^ and the above-mentioned pharmacological demonstration of LPS transport as a suitable drug target in *P. aeruginosa*^35^, provide strong support for further investigation of the druggability of the *P. aeruginosa* LptH protein.

More generally, our study highlights that experimental validation of putative essential genes, based on both *in vitro* and *in vivo* evidence, is important to confirm the results from high-throughput transposon mutagenesis, and crucial to propose novel potential molecular targets for antibacterial drug discovery programs.

## Methods

### Bacterial growth conditions

Bacterial strains used in this study are listed in Table 2. Unless otherwise stated, growth assays were performed at 37 °C in microtiter plates in MH, LB (Acumedia) or M9 minimal medium^37^ supplemented with 50 μM FeCl_3_ and either 20 mM succinate or 20 mM glucose as the carbon source. When required, arabinose was added to growth media at the indicated concentration. Bacterial growth was assessed by measuring the OD_600_ of the bacterial cultures in a Victor plate reader (Wallac). When indicated, bacterial growth was also evaluated by determining the number of CFU/ml.

### Construction of plasmids, deletion and conditional mutants

Plasmids and primers used in this study are listed in Supplementary Tables S1 and S2, respectively. In-frame deletion of each gene of interest was obtained using the *sacB*-based suicide vector pDM4 as previously described^38^. pDM4 derivatives were generated by cloning ca. 500-bp long DNA fragments corresponding to the upstream and downstream genomic regions of each gene of interest in the sequencing vector pBluescript II (Stratagene). After DNA sequencing check, the upstream and downstream fragments were excised from pBluescript II and sub-cloned into pDM4. The restriction sites used for cloning are listed in Supplementary Table S2.

Conditional mutagenesis was performed using a recently-described strategy^19^. Briefly, a mini-CTX1 derivative carrying the coding sequence of each gene of interest under the control of an arabinose-dependent regulatory element, which includes the *araC* gene, the P_BAD_ promoter and a modified ribosome binding site to reduce the basal level of expression^39^, was inserted into a neutral site of the *P. aeruginosa* genome. Then, in-frame deletion of the endogenous genes was performed by using specific pDM4 derivatives under permissive conditions (i.e. growth in the presence of 0.5% arabinose). Mini-CTX1 derivatives were generated by replacing the *tolB* gene in mini-CTX1-*araC*P_BAD_*tolB*^19^ with the coding sequence of each gene of interest. Gene deletion and insertion events were verified by PCR and DNA sequencing.

### Detergent sensitivity assay

Sensitivity to the lytic effect of SDS was assessed as previously described^19^, by determining the turbidity (OD_600_) of bacterial cell suspensions in saline after 5-min incubation at room temperature in the presence of increasing SDS concentrations (0–5%).

### *G. mellonella* infection assay

*P. aeruginosa* strains were grown in MH with 0.5% arabinose, and serial dilutions of bacterial cell suspensions in saline were injected into *G. mellonella* larvae as described^20^. Larvae were incubated at 30 °C for one week to monitor mortality. Each strain was tested in at least three independent experiments. The LD_90_ was determined using the GraphPad Prism software as previously described^40^. The number of viable cells in dead larvae was determined by plating serial dilutions of the larval hemolymph in saline on MH agar plates supplemented with 100 μg/ml of ampicillin (to which *P. aeruginosa* is intrinsically insensitive^19^) and/or 0.5% arabinose.

### Mouse lung infection model

C57Bl/6 mice (20–22 gr) were purchased from Charles River. Mice were housed in filtered cages under specific-pathogen conditions and permitted unlimited access to food and water. Prior to animal experiments, the parental strain *P. aeruginosa* PAO1 and the *lptH* and *lolA* conditional mutants were grown for 3 h to reach exponential phase in TSB with 0.5% arabinose. Next, bacterial cells were pelleted by centrifugation (2,700 × *g* for 15 min), washed twice with sterile PBS and the OD of the bacterial suspension was adjusted by spectrophotometry at 600 nm. Mice were anesthetized and the trachea directly visualized by a ventral midline incision, exposed and intubated with a sterile, flexible 22-g cannula attached to a 1 ml syringe according to established procedures^21^. A 50 μl inoculum of 10^7^ or 10^8^ CFU of PAO1, *lptH* or *lolA* were implanted via the cannula into the lung, with both lobes inoculated. After infection, mortality and body weight were monitored over one week. The animals were handled in compliance with European Communities Council Directive 86/609 for the care of laboratory animals and ethical guidelines for research in animals. All procedures were approved by the Institutional Animal Care and Use Committee (IACUC) of the San Raffaele Scientific Institute (Milan, Italy) and adhered strictly to the Italian Ministry of Health guidelines for the use and care of experimental animals.

### Bioinformatics predictions

Protein subcellular localization, presence of signal peptide for protein export and of transmembrane helices (TMHs) were predicted with the PSORTb Subcellular Localization Prediction Tool (www.psort.org/psortb/), the SignalP 4.1 Server (http://www.cbs.dtu.dk/services/SignalP/) and the TMHMM Server 2.0 (http://www.cbs.dtu.dk/services/TMHMM/), respectively.

### Statistical analysis

Two-way ANOVA with Bonferroni’s multiple comparison test was used to compare change in body weight. Survival curves for the mouse infection assay were analyzed using the log-rank Mantel–Cox test. Statistical analysis was performed with the software GraphPad.

## Additional Information

**How to cite this article**: Fernández-Piñar, R. *et al. In vitro* and *in vivo* screening for novel essential cell-envelope proteins in *Pseudomonas aeruginosa*. *Sci. Rep.* **5**, 17593; doi: 10.1038/srep17593 (2015).

## Supplementary Material

Supplementary Information

## Figures and Tables

**Table 1:** Selected candidate essential genes of *P. aeruginosa* PAO1 investigated in this study.

PAO1 ID	Gene name	PA14 ortholog	Signal peptide^1^	Transmembrane helix (TMH)^2^	Subcellular localization^3^	Protein function	Gene length (bp)
PA0517	*nirC*	PA14_06730	Yes	No	Periplasm	C-type cytochrome precursor	360
PA1645		PA14_43230	Yes	No	Non-cytoplasmic^4^	Hypothetical protein	408
PA1981		PA14_38880	Yes	No	Extracellular	Hypothetical protein	648
PA2614	*lolA*	PA14_30310	Yes	No	Periplasm	Periplasmic chaperone LolA	627
PA3786		PA14_15110	No	1 (N-terminal, in → out orientation)^5^	IM	Hypothetical protein	390
PA4460	*lptH*	PA14_57920	Yes	No	Periplasm	LPS transport protein LptA	527
PA4485		PA14_58210	Yes^6^	No	OM^7^	Conserved hypothetical protein	378
PA5126		PA14_67700	No	1 (N-terminal, in → out orientation)^5^	IM	Hypothetical protein	468

^1^Predicted by SignalP 4.1 (http://www.cbs.dtu.dk/services/SignalP/).

^2^Predicted by TMHMM Server 2.0 (http://www.cbs.dtu.dk/services/TMHMM/).

^3^Predicted by PSORTb (http://www.psort.org/psortb/). Abbreviations: OM, outer membrane; IM, inner membrane.

^4^According to PSORTb, this protein has an equal probability of having an IM, periplasmic, OM or extracellular localization.

^5^This protein is predicted to be anchored to the IM by means of a single N-terminal TMH, with the bulk of the protein exposed to the periplasmic space.

^6^Putative lipoprotein according to LipoP 1.0 Server (http://www.cbs.dtu.dk/services/LipoP/).

^7^The OM localization of this putative lipoprotein was predicted by ref. 30.

**Table 2:** Bacterial strains used in this study.

Strain	Genotype and/or relevant characteristics	Reference/source
*P. aeruginosa*		
PAO1 (ATCC15692)	Prototroph	American type culture collection
PAO1 ΔPA0517	PAO1 with an in-frame deletion of the PA0517 gene	This study
PAO1 ΔPA1645	PAO1 with an in-frame deletion of the PA1645 gene	This study
PAO1 ΔPA1981	PAO1 with an in-frame deletion of the PA1981 gene	This study
PAO1 ΔPA3786	PAO1 with an in-frame deletion of the PA3786 gene	This study
PAO1 ΔPA4485	PAO1 with an in-frame deletion of the PA4485 gene	This study
PAO1 ΔPA5126	PAO1 with an in-frame deletion of the PA5126 gene	This study
PAO1 *araC*P_BAD_*lolA* Δ*lolA*	PAO1 with an arabinose-inducible copy of *lolA* (PA2614) inserted into the *attB* neutral site and an in-frame deletion of the endogenous copy of *lolA*	This study
PAO1 *araC*P_BAD_*lptH* Δ*lptH*	PAO1 with an arabinose-inducible copy of *lptH* (PA4460) inserted into the *attB* neutral site and an in-frame deletion of the endogenous copy of *lptH*	This study
*E. coli*		
S17.1λ*pir*	*thi pro hsdR hsdM*^*+*^ *recA RP4-2-Tc::Mu-Km::Tn7 λpir,* Gm^R^	41
DH5αF’	*recA1 endA1 hsdR17 supE44 thi-1 gyrA96 relA1* Δ(*lacZYA-argF*)U169[ϕ*80 dlacZ*Δ*M15*], Nal^R^	42

**Table 3:** Lethal dose 90% (LD_90_) in *G. mellonella* larvae for *P. aeruginosa* PAO1 and the isogenic deletion or conditional mutants analysed in this study^1^.

Strain	LD_90_	R^2^
PAO1	2.7	0.71
PAO1 ΔPA0517	2.3	0.78
PAO1 ΔPA1645	3.2	0.88
PAO1 ΔPA1981	6.8	0.73
PAO1 ΔPA3786	5.0	0.83
PAO1 ΔPA4485	4.8	0.78
PAO1 ΔPA5126	4.1	0.79
PAO1 *araC*P_BAD_*lolA* Δ*lolA*	63.4	0.85
PAO1 *araC*P_BAD_*lptH* Δ*lptH*	1.6 × 10^7^	0.73

^1^The LD_90_ and R^2^ values were determined using the GraphPad Prism software and the survival curves shown in Fig. 3 or Supplementary Fig. S4.
