# Supplementary material for: In vitro and in vivo screening for novel essential cell-envelope proteins in Pseudomonas aeruginosa
Source: Sci Rep. 2015 Dec 1;5:17593. doi: 10.1038/srep17593 (PMC4665194; doi:10.1038/srep17593)
Supplement: Supplementary Information [file srep17593-s1.pdf]

SUPPLEMENTARY INFORMATION

In vitro and *in vivo* screening for novel essential cell-envelope proteins in *Pseudomonas aeruginosa*

Regina Fernández-Piñar, Alessandra Lo Sciuto, Alice Rossi, Serena Ranucci, Alessandra Bragonzi, and Francesco Imperi

Table S1. Plasmids used in this study.

Plasmid	Description	Reference/source
pBluescript II KS+	Cloning vector; ColE1 replicon; Ap^R	Stratagene
pDM4	Suicide vector; *sacBR*, *oriR6K*; Cm^R	[38]
pDM4ΔPA0517	pDM4 derivative for PA0517 in-frame deletion	This study
pDM4ΔPA1645	pDM4 derivative for PA1645 in-frame deletion	This study
pDM4ΔPA1981	pDM4 derivative for PA1981 in-frame deletion	This study
pDM4ΔPA2614	pDM4 derivative for PA2614 (*lolA*) in-frame deletion	This study
pDM4ΔPA3786	pDM4 derivative for PA3786 in-frame deletion	This study
pDM4ΔPA4460	pDM4 derivative for PA4460 (*lptH*) in-frame deletion	This study
pDM4ΔPA4485	pDM4 derivative for PA4485 in-frame deletion	This study
pDM4ΔPA5126	pDM4 derivative for PA5126 in-frame deletion	This study
mini-CTX1	Self-proficient integration vector with *tet*, Ω-*FRT*-*attP*-MCS, *ori*, *int*, and *oriT*; Tc^R	[43]
mini-CTX1-*araC*P_{BAD}*tolB*	mini-CTX1 derivative carrying *araC*P_{BAD}*tolB*	[19]
mini-CTX1-*araC*P_{BAD}PA2614	mini-CTX1-*araC*P_{BAD}*tolB* derivative in which *tolB* has been replaced with PA2614 (*lolA*)	This study
mini-CTX1-*araC*P_{BAD}PA4460	mini-CTX1-*araC*P_{BAD}*tolB* derivative in which *tolB* has been replaced with PA4460 (*lptH*)	This study

Additional reference (not included in the main text):

43. Hoang, T. T., Kutchma, A. J., Becher, A. & Schweizer, H. P. Integration-proficient plasmids for *Pseudomonas aeruginosa*: site-specific integration and use for engineering of reporter and expression strains. Plasmid **43,** 59-72 (2000).

Table S2. Primers used in this study [a]

Primer name	Sequence (5’→3’)[b]	Restriction site	Application
PA2614_FW	CCCGATATCGATGCGACTGATCCGCAC	EcoRV	Generation of mini-CTX1-*araC*P_{BAD}PA2614
PA2614_RV	CGGAATTCTTACTCCTGGATCACGTCG	EcoRI	Generation of mini-CTX1-*araC*P_{BAD}PA2614
PA2614mut_UP_FW	CCGCTCGAGGGGGCGCCGAACAAC	*XhoI*	Generation of pDM4ΔPA2614
PA2614mut_UP_RV	CGGGATCCCATGGCCAGGGCGGC	*BamHI*	Generation of pDM4ΔPA2614
PA2614mut_DOWN_FW	CGGGATCCTTCGACGTTCCGCCGG	*BamHI*	Generation of pDM4ΔPA2614
PA2614mut_DOWN_RV	GCTCTAGACATACACGCGAGCCCG	*XbaI*	Generation of pDM4ΔPA2614
PA4460_FW	CCCAAGCTTATGAGGTTCGTTAATACCCTC	HindIII	Generation of mini-CTX1-*araC*P_{BAD}PA4460
PA4460_RV	CGGAATTCTGGGCTTTGAGCGTTGCC	EcoRI	Generation of mini-CTX1-*araC*P_{BAD}PA4460
PA4460mut_UP_FW	ACGCGTCGACTACTACTGGAACGTCGGG	*SalI*	Generation of pDM4ΔPA4460
PA4460mut_UP_RV	CGGGATCCGAGGGCGGCAGTCAG	*BamHI*	Generation of pDM4ΔPA4460
PA4460mut_DOWN_FW	CGGGATCCCAGTAATGGCAACGCTC	*BamHI*	Generation of pDM4ΔPA4460
PA4460mut_DOWN_RV	GCTCTAGATCTGCTTGATGTCGCCG	*XbaI*	Generation of pDM4ΔPA4460
PA0517mut_UP_FW	CGGCTCGAGTACAACAAGCGCGGC	*XhoI*	Generation of pDM4ΔPA0517
PA0517mut_UP_RV	CGGGATCCAAGGCGTGGCTGGCGG	*BamHI*	Generation of pDM4ΔPA0517
PA0517mut_DOWN_FW	CGGGATCCCGGCTGGCTGGTGGAC	*BamHI*	Generation of pDM4ΔPA0517
PA0517mut_DOWN_RV	GCTCTAGAAGTCGGCGATCCAGATC	*XbaI*	Generation of pDM4ΔPA0517
PA1645mut_UP_FW	CGGCTCGAGCACCTGCTCCACCTCG	*XhoI*	Generation of pDM4ΔPA1645
PA1645mut_UP_RV	CGGGATCCAGCAACAGCGGCAATACG	*BamHI*	Generation of pDM4ΔPA1645
PA1645mut_DOWN_FW	CGGGATCCGGTCGACGAGTGCCTGC	*BamHI*	Generation of pDM4ΔPA1645
PA1645mut_DOWN_RV	GCTCTAGAGGGCGCCAGCAACCTG	*XbaI*	Generation of pDM4ΔPA1645
PA1981mut_UP_FW	CCCTTCGTCGACAACATCAC	*SalI*	Generation of pDM4ΔPA1981
PA1981mut_UP_RV	CGGGATCCGGCGCCGAGCAGGG	*BamHI*	Generation of pDM4ΔPA1981
PA1981mut_DOWN_FW	CGGGATCCAAGGATTGCCCCGCGGG	*BamHI*	Generation of pDM4ΔPA1981
PA1981mut_DOWN_RV	GCTCTAGAAGCCTTGCAGGCGGTG	*XbaI*	Generation of pDM4ΔPA1981
PA3786mut_UP_FW	CCGCTCGAGGGTGATGCGCTGCTCG	*XhoI*	Generation of pDM4ΔPA3786
PA3786mut_UP_RV	CGGGATCCAGCCAGGCCACGGGG	*BamHI*	Generation of pDM4ΔPA3786
PA3786mut_DOWN_FW	CGGGATCCTGTCGCCTGACTCCTC	*BamHI*	Generation of pDM4ΔPA3786
PA3786mut_DOWN_RV	GCTCTAGATTTCCACCGGCACGTCG	*XbaI*	Generation of pDM4ΔPA3786
PA4485mut_UP_FW	ACGCGTCGACTACCGTTACTTCCCC	*SalI*	Generation of pDM4ΔPA4485

PA4485mut_UP_RV	CGGGATCCGGCGGATGGGGTGGGC	*BamHI*	Generation of pDM4ΔPA4485
PA4485mut_DOWN_FW	CGGGATCCAGCGGCGTGGCGCCG	*BamHI*	Generation of pDM4ΔPA4485
PA4485mut_DOWN_RV	GCTCTAGACCTGGTCACCGAAGAGGG	*XbaI*	Generation of pDM4ΔPA4485
PA5126mut_UP_FW	ACGCGTCGACGCCGTCGGTCCAG	*SalI*	Generation of pDM4ΔPA5126
PA5126mut_UP_RV	CGGGATCCGGAGCCCTGCATGTTC	*BamHI*	Generation of pDM4ΔPA5126
PA5126mut_DOWN_FW	CGGGATCCGACGGCCGGGAATAAAAG	*BamHI*	Generation of pDM4ΔPA5126
PA5126mut_DOWN_RV	GCTCTAGACCGTCCGCAGCACAGCC	*XbaI*	Generation of pDM4ΔPA5126

[a] All PCRs were performed using the genomic DNA of *P. aeruginosa* PAO1 as the template.
[b] The restriction site used for cloning is underlined in the primer sequence.

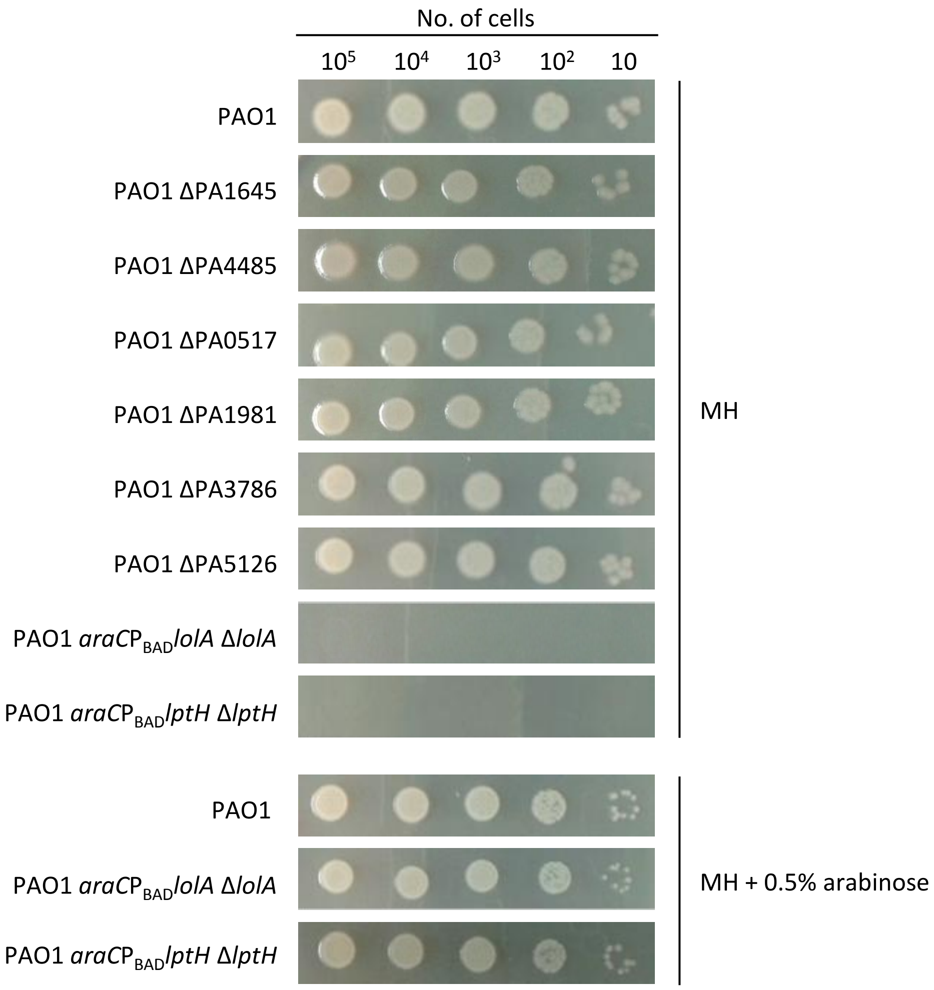

Figure S1. Growth of *P. aeruginosa* PAO1 and all the deletion or conditional mutants generated in this study on MH agar plates supplemented or not with 0.5% arabinose. For each strain, 5 µl of 10-fold serial dilutions of exponential phase cultures in MH (or MH with 0.5% arabinose in the case of conditional mutants), ranging from ca. 10^5 to 10 viable cells, were spotted onto the plates, that were then incubated for 20 h at 37°C. The images are representative of three independent experiments giving comparable results.

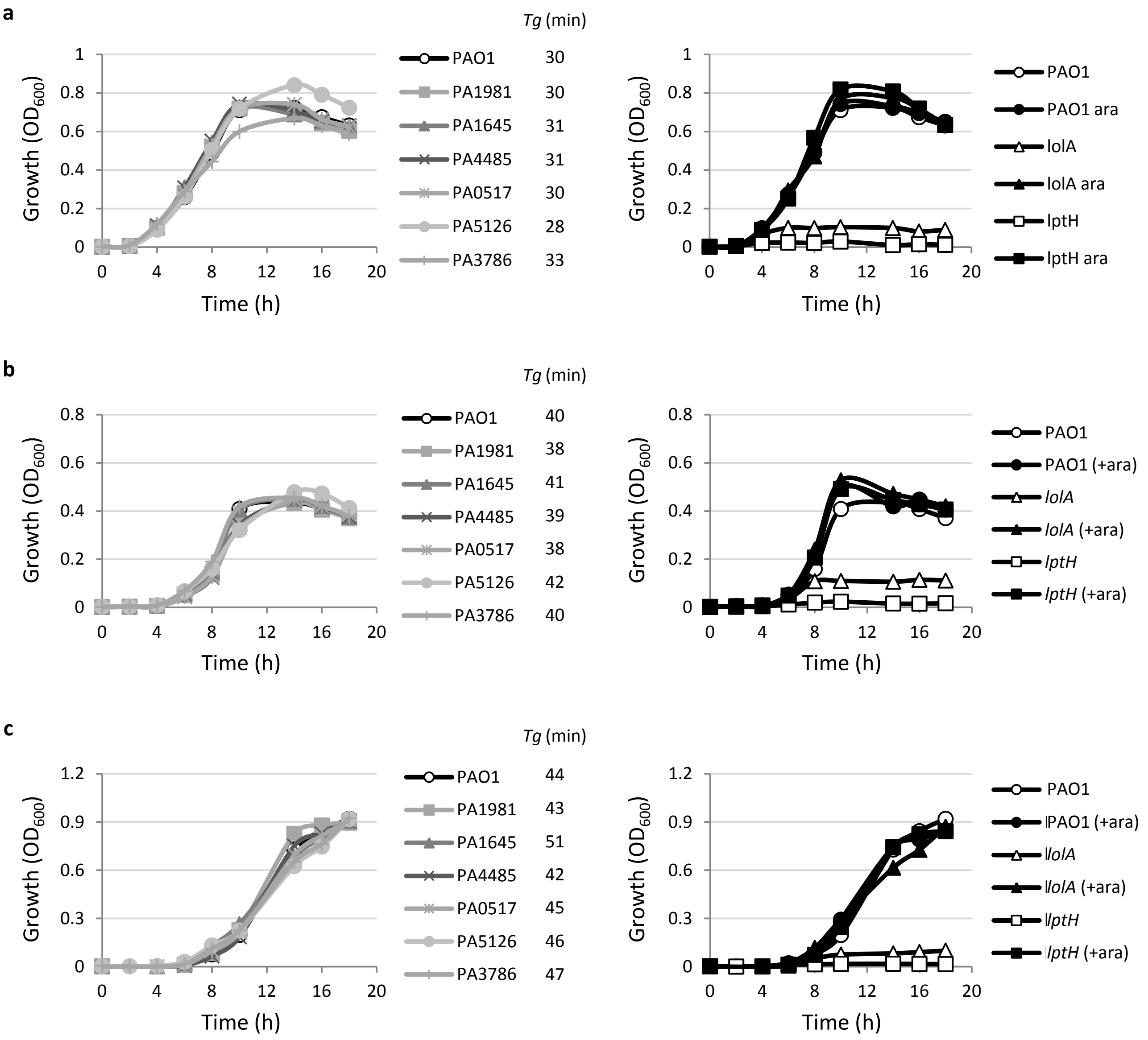

Figure S2. Growth curves of the wild type strain PAO1 and the PA0517, PA1645, PA1981, PA3786, PA4485 and PA5126 deletion mutants (left panels) or the *lptH* and *lolA* conditional mutants (right panels) in LB broth (**a**) or in M9 minimal medium supplemented with either succinate (**b**) or glucose (**b**) as carbon sources, at 37°C in microtiter plates at 200 rpm. Conditional mutants were cultured in the absence or in the presence of 0.5% arabinose (+ara). Generation times (*Tg*) for the wild type strain and each deletion mutant are reported in the figure. Results are the mean of two independent experiments performed in triplicate, with standard deviations (SD) being < 11% of the values.

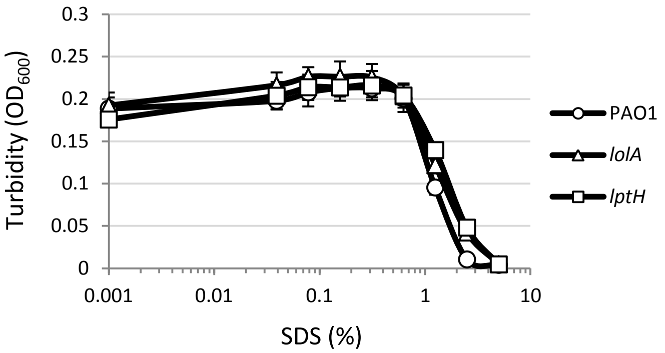

Figure S3. Lytic effect of different SDS concentrations (0-5%), measured as decrease in cell suspension turbidity (OD_{600}), on the PAO1 wild type and the *lptH* and *lolA* conditional mutant grown to late-exponential phase in MH supplemented with 0.5% arabinose. Results are the mean (± SD) of two independent experiments performed in duplicate.

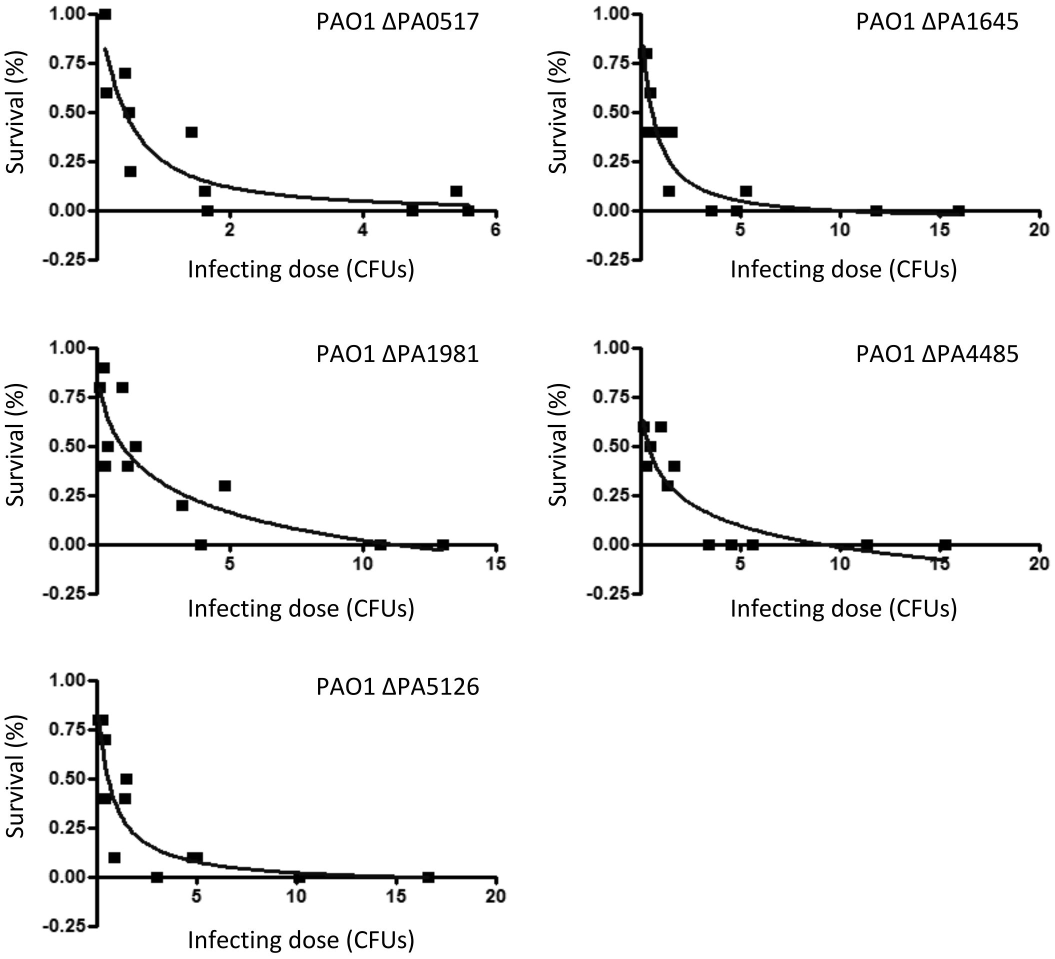

Figure S4. Survival curves, generated by the GraphPad Prism software, of *G. mellonella* larvae infected with different doses of the deletion mutants PAO1 ΔPA0517, PAO1 ΔPA1645, PAO1 ΔPA1981, PAO1 ΔPA4485 and PAO1 ΔPA5126.

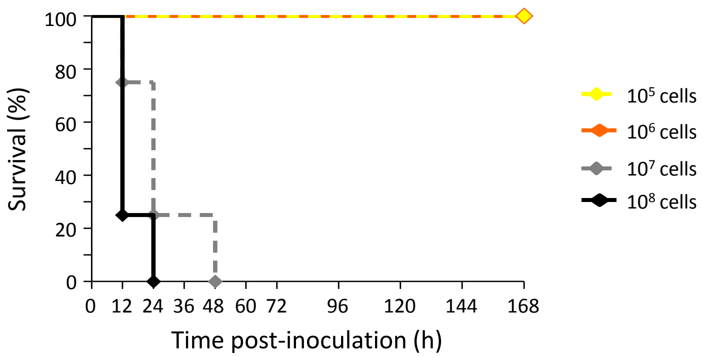

Figure S5. Mortality curves for mice (n = 4) infected with 10^5, 10^6, 10^7 or 10^8 cells of the wild type strain *P. aeruginosa* PAO1.

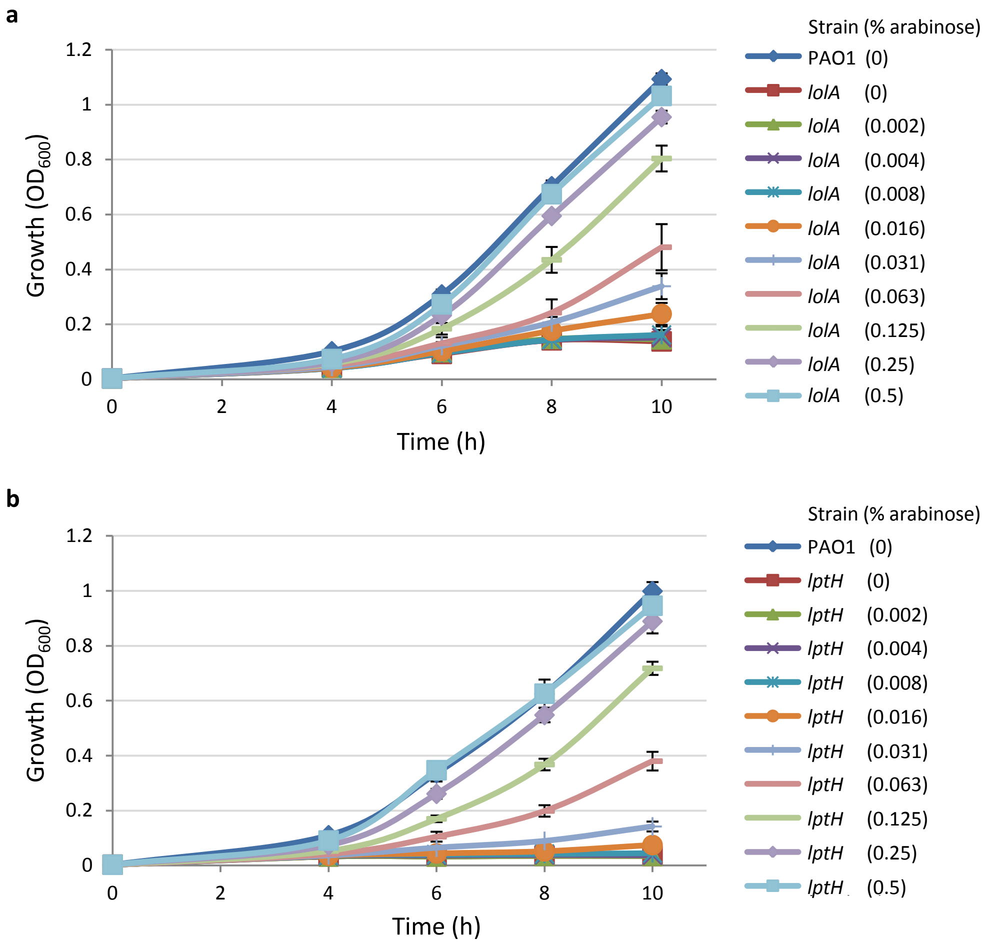

Figure S6. Growth of PAO1 and (a) the *lolA* conditional mutant or (b) the *lptH* conditional mutant in microtiter plates at 200 rpm in MH broth supplemented with increasing concentrations of arabinose (0-0.5%) after 14 h at 37°C. Results are the mean (± SD) of two independent experiments performed in duplicate.
